# An Incidental Finding of a Glucagon-Like Peptide 1 (GLP-1)-Induced Acute Kidney Injury: A Case Report

**DOI:** 10.7759/cureus.45261

**Published:** 2023-09-14

**Authors:** Andy Aleman Espino, Erik Aleman Espino, Claudia Aleman Oliva, Hamlet Monteagudo, Odalys Frontela

**Affiliations:** 1 Osteopathic Medicine, Nova Southeastern University Dr. Kiran C. Patel College of Osteopathic Medicine, Fort Lauderdale, USA; 2 Internal Medicine, Larkin Community Hospital Palm Springs Campus, Hialeah, USA; 3 Internal Medicine, Larkin Community Hospital, Hialeah, USA

**Keywords:** acute kidney injury, clinical case report, glp-1 receptor agonist, general nephrology, acute interstitial nephritis (ain)

## Abstract

Acute kidney injury (AKI) involves a rapid decline in kidney function, classified into prerenal, intrarenal, and postrenal causes. Drug-induced AKI's complex pathophysiology includes altered hemodynamics, inflammation, crystal deposition, hemolysis, and rhabdomyolysis. This report details a 42-year-old female with hypertension and diabetes who, following a dog bite, exhibited reduced kidney function (GFR: 16 ​​mL/min/1.73m^2^; BUN/Cr: 23/3.23 mg/dL). A renal ultrasound revealed no stones or masses, and the recent use of tirzepatide was identified. Discontinuation of the drug, IV fluid maintenance, and close monitoring led to swift kidney function improvement. This case underscores the importance of recognizing drug-induced AKI, even in unrelated complaints, and highlights the need for vigilance and research into the adverse effects of medications such as glucagon-like peptide 1 (GLP-1) receptor agonists.

## Introduction

This case report delineates an incidental discovery of drug-induced acute kidney injury (AKI) in a patient who presented to the emergency department, primarily because of a chief complaint of a dog bite.

AKI is defined as the acute and fast decline in kidney function seen by a decrease in glomerular filtration rate (GFR). Other often-seen findings include increased blood urea nitrogen (BUN) and decreased creatinine clearance. However, these values can stay within the normal range in the first days following the kidney insult. Patients also often present with decreased urine output [1].

The possible etiologies of AKI are subdivided into three general categories that include prerenal, intrarenal, and postrenal causes. Most cases, approximately 50%, are attributed to prerenal insults caused by a decrease in arterial blood pressure, as in the case of heart failure, fluid or blood loss, sepsis, etc. [2].

Intrarenal etiologies account for around 45% of AKI cases and encompass acute tubulointerstitial nephritis, acute vasculopathy, and acute tubular necrosis. These may arise from the use of certain drugs (NSAIDs, beta-lactams, allopurinol), infections (leptospirosis, EBV), and electrolyte and metabolic disorders [2].

Finally, approximately 5% of AKI cases are attributed to postrenal issues, primarily urinary tract obstruction. The underlying causes of postrenal AKI include hematoma of the renal pelvis, malignancies, and neurological disorders that lead to bladder dysfunction [2].

AKI diagnosis follows KDIGO guidelines, which categorize AKI based on how well the kidneys are functioning. These criteria include a 50% increase in serum creatinine (SCr) within seven days, a rise in SCr by 0.3 mg/dl (26.5 mmol/l) within two days, or the presence of oliguria [3].

The pathophysiology of drug-induced AKI is complicated, involving kidney damage resulting from altered intraglomerular hemodynamics, inflammation, crystal deposition, and rhabdomyolysis, among other factors. Hospitalized patients commonly experience AKI due to NSAIDs, aminoglycoside antibiotics, contrast agents, and angiotensin-converting enzyme inhibitors (ACEIs). Additionally, crystal-induced nephropathy is associated with acyclovir, ganciclovir, ampicillin, sulfonamides, methotrexate, and ciprofloxacin use. Statins and alcohol can lead to AKI because of their toxic effect on myocyte function, leading to rhabdomyolysis. Furthermore, drugs that impair mitochondrial function, increase free radicals and oxidative stress, and interfere with tubular transport are responsible for acute interstitial nephritis (AIN) and tubular cell toxicity. Examples include amphotericin B, aminoglycosides, cisplatin, rifampin, and beta-lactams, among others [4].

Patients with AIN typically present asymptomatic, or with no specific symptoms. Most cases are non-oliguric, though oliguria may be observed. The onset of renal failure varies from weeks to months after exposure to the offending drug. Drug-induced AIN can exhibit an allergic-like course, characterized by the presence of a skin rash, eosinophilia, and low-grade fever, but the simultaneous manifestation of all three symptoms together is uncommon. Laboratory findings often include sterile pyuria and sub-nephrotic range proteinuria in about 82% and 93% of patients, respectively. Hematuria, as observed under microscopy, has been reported in approximately 67% of patients. Despite peripheral eosinophilia being associated with 35% of drug-induced AIN cases, its sensitivity and specificity as a diagnostic marker are limited. For a definitive diagnosis, a kidney biopsy may be necessary [5].

Although relatively uncommon, case reports of glucagon-like peptide 1 (GLP-1) receptor agonists associated with AKI have been documented. The increased use of drugs like tirzepatide, semaglutide, or dulaglutide (GLP-1 receptor agonists) in the treatment of diabetes and off-label for weight loss emphasizes the importance for medical providers to be aware of potential side effects. As such, further research and monitoring are warranted to better understand and manage this rare occurrence [6,7].

## Case presentation

A 42-year-old female with a past medical history of hypertension (HTN) and diabetes mellitus (DM) presented to our emergency room (ER) with a dog bite wound on her right hand. The incident had occurred the previous night. At the ER, the patient was alert, awake, and oriented (AAOx3: person, time, and place) in non-acute distress but complained of severe pain. On inspection, the hand was swollen and tender to the touch, and the range of motion was limited. A review of the systems was negative for any associated fever, nausea, vomiting, shortness of breath, chest, or back pain. Vital signs were unremarkable. Laboratory work performed after the incident in an urgent care outpatient clinic showed a significantly high white blood cell count (WBC) of 16,000 WBC/mcL (normal: 4,000-11,000 WBC/mcL) and a C-reactive protein (CRP) of >15 mg/L (normal: 1-3 mg/L). In the ER, blood cultures were taken, and she was started on antibiotics (azithromycin and clindamycin) and hydromorphone for pain control. The patient was admitted under the care of our Internal Medicine team for further management and observation because of the severity of the dog bite, which was exacerbated by the presence of infection.

On the first day of admission, the laboratory workup was significant for low potassium (K) levels of 3.2 mmol/L (repeat K order was 2.7 mmol/L) (normal: 3.5-5 mmol/L), increased BUN/Cr of 23/3.23 mg/dL (normal: 7-18/0.6-1.2 mg/dL), and GFR of 16 mL/min/1.73m^2 ^(normal: 90-120 mL/min/1.73 m^2^). Urinalysis was unremarkable. The medicine team started the patient on 40mEq of oral K and normal saline intravenous fluid (NS IV fluid). The patient denied any history of kidney disease. Laboratories performed the prior month showed no evidence of kidney injury, with Cr levels of 0.49 mg/dL and GFR of 121 mL/min/1.73 m^2^. In a further interview, the patient disclosed that tirzepatide was recently added to her regular prescriptions (losartan/HCTZ, amlodipine, and labetalol) to control her type II DM.

**Figure 1:**
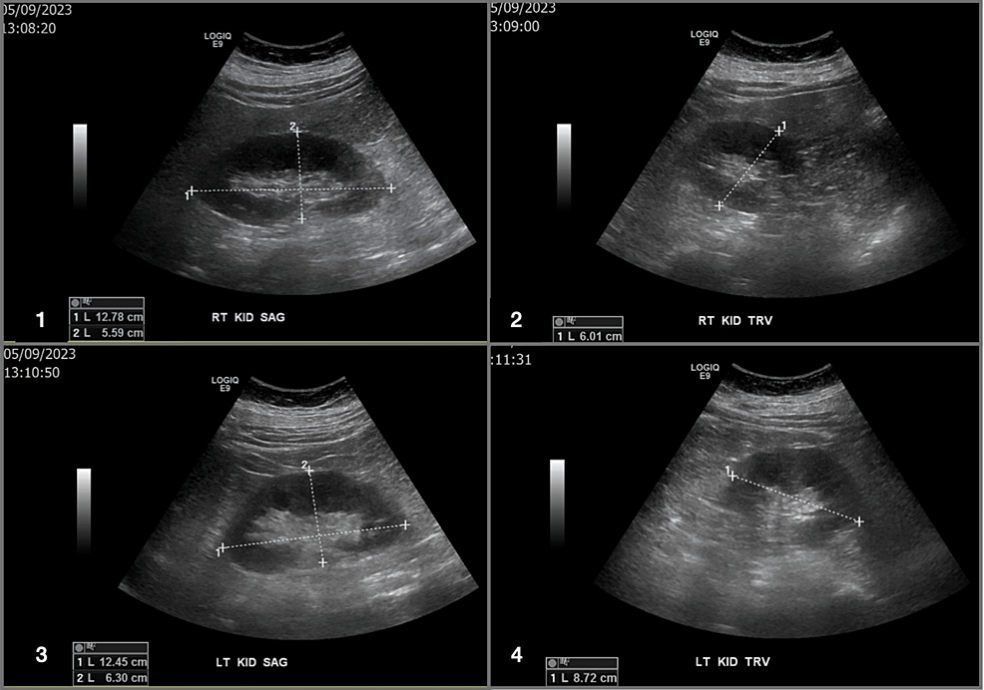
Right and Left Renal Ultrasound. 1- Right Kidney Sagittal View 2- Right Kidney Transverse View 3- Left Kidney Sagital View 4- Left Kidney Transverse View

**Figure 2:**
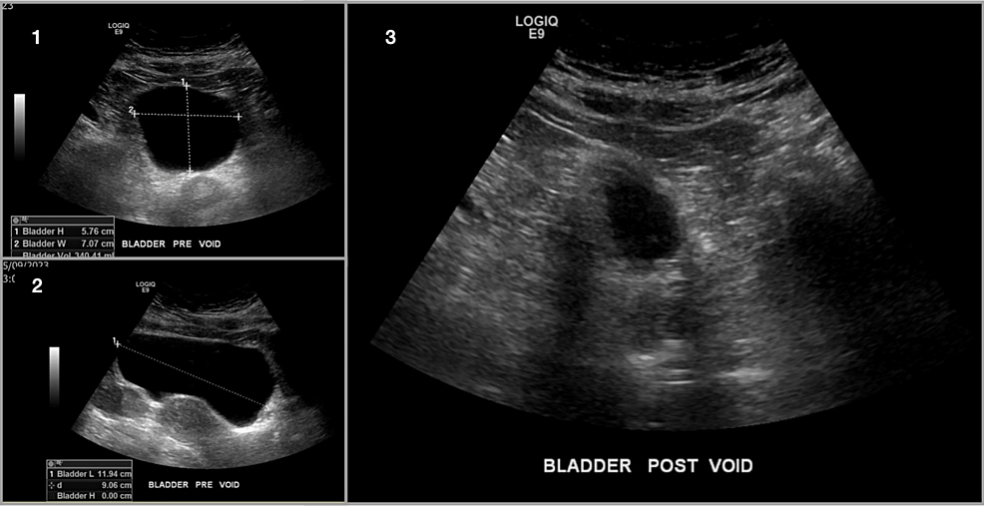
Bladder Ultrasound. 1- Bladder Pre-Void Sagittal View 2- Bladder Pre-Void Transverse View 3- Bladder Post-Void

A renal ultrasound (US) showed no sonographic evidence of renal stones, masses, or hydronephrosis. Figure 1 shows that the patient was able to empty her bladder completely. Figure 2 shows that the US was only remarkable for an incidental asymptomatic right ovarian cyst.

The nephrology team was consulted, and they suspected that the patient was suffering from AIN most likely due to the recent introduction of the GLP-1 receptor agonist tirzepatide to her scheduled prescriptions. They discontinued the medication, continued NS infusion at 100 ml/hr, initiated Ferric gluconate 125 mg/day x eight doses, and continued monitoring renal function. They also recommended adjusting the patient’s medications and avoiding NSAIDs, ACE-Is, ARBs, and other nephrotoxic agents.

On the second day after admission, her kidney function had significantly improved (BUN: 16 mg/dL; creatinine: 1.83 mg/dL, and GFR: 30 mL/min/1.73 m²), WBC count had normalized, and there was no clinical evidence of infection. Both the nephrology team and the infectious disease team recommended her discharge. The patient was discharged home on oral antibiotics (azithromycin) with recommendations to follow up with her primary physician within 48 hours. Our ER team also made a follow-up call to the patient a couple of days after her discharge, during which she reported no complaints. She informed us that she had already scheduled an appointment with her primary physician for further follow-up care.

## Discussion

This case report describes a patient who presented to the emergency department with a dog bite wound but was incidentally found to have AKI. AKI is characterized by a rapid decline in kidney function, often indicated by a decrease in GFR, increased BUN, and decreased creatinine clearance. In this patient, laboratory findings showed an increased BUN/Cr and reduced GFR, suggestive of AKI [1].

The possible etiologies of AKI were thoroughly considered, with a focus on drug-induced AKI due to the recent introduction of tirzepatide alongside the patient's regular medications for diabetes. The literature underscores the association between various drugs and AKI, encompassing NSAIDs, aminoglycoside antibiotics, contrast agents, ACEIs, and other nephrotoxic agents. Additionally, crystal-induced nephropathy has been linked to specific medications like acyclovir, ganciclovir, and sulfonamides [2]. Notably, the decision to discontinue potentially nephrotoxic drugs like losartan/HCTZ was made after the patient's hospital presentation, as the precise causative agent was initially uncertain. It is crucial to emphasize that the patient had no history of kidney disease, and her kidney function had remained unremarkable for years while on losartan/HCTZ. Moreover, laboratory work conducted during a routine primary care physician (PCP) visit just one month before her hospital admission and immediately before initiating tirzepatide indicated normal kidney function. This temporal correlation strongly supports the notion that the introduction of tirzepatide played a significant role in the development of the suspected AKI.

Further investigation into the patient's clinical presentation revealed signs of AIN, including sterile pyuria and signs of acute renal failure. Notably, the patient did not present with an allergic-like course (e.g., skin rash, eosinophilia, or low-grade fever) and had no kidney-related symptoms [5]. However, the nephrology team considered the patient's medication history, laboratory findings, and clinical symptoms, leading to the diagnosis of drug-induced AIN as a plausible cause of AKI.

The management of AIN involves promptly discontinuing the offending drug and initiating early corticosteroid use, as done in this case. Corticosteroids have been associated with improved renal recovery in patients with AIN [5].

The study by Ohtake et al. (2017) also highlights different adverse side effects of GLP-1 receptor agonists including AKI. With the increasing use of GLP-1 receptor agonists like tirzepatide in diabetes and weight loss treatments, it becomes vital for medical providers to be aware of potential side effects, including AKI. Further research and monitoring of such medications are warranted to ensure their safe use and to identify and manage adverse reactions promptly [8].

## Conclusions

This case report underscores the crucial role of vigilance in identifying drug-induced AKI, particularly in cases where seemingly unrelated complaints, such as a dog bite wound, are presented. The patient's medical history, recent medication adjustments, and clinical manifestation were all pivotal in pinpointing drug-induced AIN as the underlying cause of AKI in this scenario. Healthcare practitioners must remain attentive to the diverse range of medications capable of inducing AKI and should contemplate this prospect when confronted with patients exhibiting AKI. Timely recognition and intervention in drug-induced AIN, encompassing the swift cessation of the implicated drug and initiation of corticosteroid therapy, can significantly enhance renal outcomes. Further investigation and continuous monitoring of GLP-1 receptor agonists, including agents like tirzepatide, are imperative, particularly given the escalating utilization of these drugs in diabetes and weight management interventions. Enhancing comprehension of potential adverse effects will empower medical professionals to ensure the judicious administration of such treatments and to promptly detect and manage any unfavorable reactions.

## References

[1] Goyal A, Daneshpajouhnejad P, Hashmi MF, Bashir K (2023). Acute kidney injury. StatPearls.

[2] Patschan D, Müller GA (2015). Acute kidney injury. J Inj Violence Res.

[3] CKD Work Group (2013). KDIGO 2012 clinical practice guideline for the evaluation and management of chronic kidney disease. Kidney Int Suppl.

[4] Ghane Shahrbaf F, Assadi F (2015). Drug-induced renal disorders. J Renal Inj Prev.

[5] Naik RH, Annamaraju P (2023). Interstitial nephritis. StatPearls [Internet].

[6] Narayana SK, Talab S, Elrishi M (2012). Liraglutide-induced acute kidney injury. Pract Diabetes.

[7] Chaiban J, Obeid M, Patel R (2022). Abstract # 1161911: acute renal failure: a rare and uncommon side effect of semaglutide. Endocr Pract.

[8] Filippatos TD, Panagiotopoulou TV, Elisaf MS (2014). Adverse effects of GLP-1 receptor agonists. Rev Diabet Stud.

